# Therapeutic Perspectives on the Modulation of G-Protein Coupled Estrogen Receptor, GPER, Function

**DOI:** 10.3389/fendo.2020.591217

**Published:** 2020-11-23

**Authors:** Milad Rouhimoghadam, Anh S. Lu, Aliasger K. Salem, Edward J. Filardo

**Affiliations:** ^1^ Department of Surgery, University of Iowa, Carver College of Medicine, Iowa City, IA, United States; ^2^ Holden Comprehensive Cancer Center, University of Iowa, Iowa City, IA, United States; ^3^ College of Pharmacy, University of Iowa, Iowa City, IA, United States

**Keywords:** GPER, estrogen receptors, therapeutics, anti-estrogens, cancer

## Abstract

Estrogens exert their physiological and pathophysiological effects *via* cellular receptors, named ERα, ERβ, and G-protein coupled estrogen receptor (GPER). Estrogen-regulated physiology is tightly controlled by factors that regulate estrogen bioavailability and receptor sensitivity, while disruption of these control mechanisms can result in loss of reproductive function, cancer, cardiovascular and neurodegenerative disease, obesity, insulin resistance, endometriosis, and systemic lupus erythematosus. Restoration of estrogen physiology by modulating estrogen bioavailability or receptor activity is an effective approach for treating these pathological conditions. Therapeutic interventions that block estrogen action are employed effectively for the treatment of breast and prostate cancer as well as for precocious puberty and anovulatory infertility. Theoretically, treatments that block estrogen biosynthesis should prevent estrogen action at ERs and GPER, although drug resistance and ligand-independent receptor activation may still occur. In addition, blockade of estrogen biosynthesis does not prevent activation of estrogen receptors by naturally occurring or man-made exogenous estrogens. A more complicated scenario is provided by anti-estrogen drugs that antagonize ERs since these drugs function as GPER agonists. Based upon its association with metabolic dysregulation and advanced cancer, GPER represents a therapeutic target with promise for the treatment of several critical health concerns facing Western society. Selective ligands that specifically target GPER have been developed and may soon serve as pharmacological agents for treating human disease. Here, we review current forms of estrogen therapy and the implications that GPER holds for these therapies. We also discuss existing GPER targeted drugs, additional approaches towards developing GPER-targeted therapies and how these therapies may complement existing modalities of estrogen-targeted therapy.

## Introduction

This review is organized in three general sections. First, we review basic information regarding estrogen bioavailability and its receptors. Second, we discuss the impact that GPER has upon our understanding of the influence of estrogen on human disease, and its implications for anti-estrogen therapy. Finally, we review existing pharmacological compounds that selectively target GPER and outline future potential approaches for targeting GPER.

## Estrogen and Its Receptors

**Figure 1:**
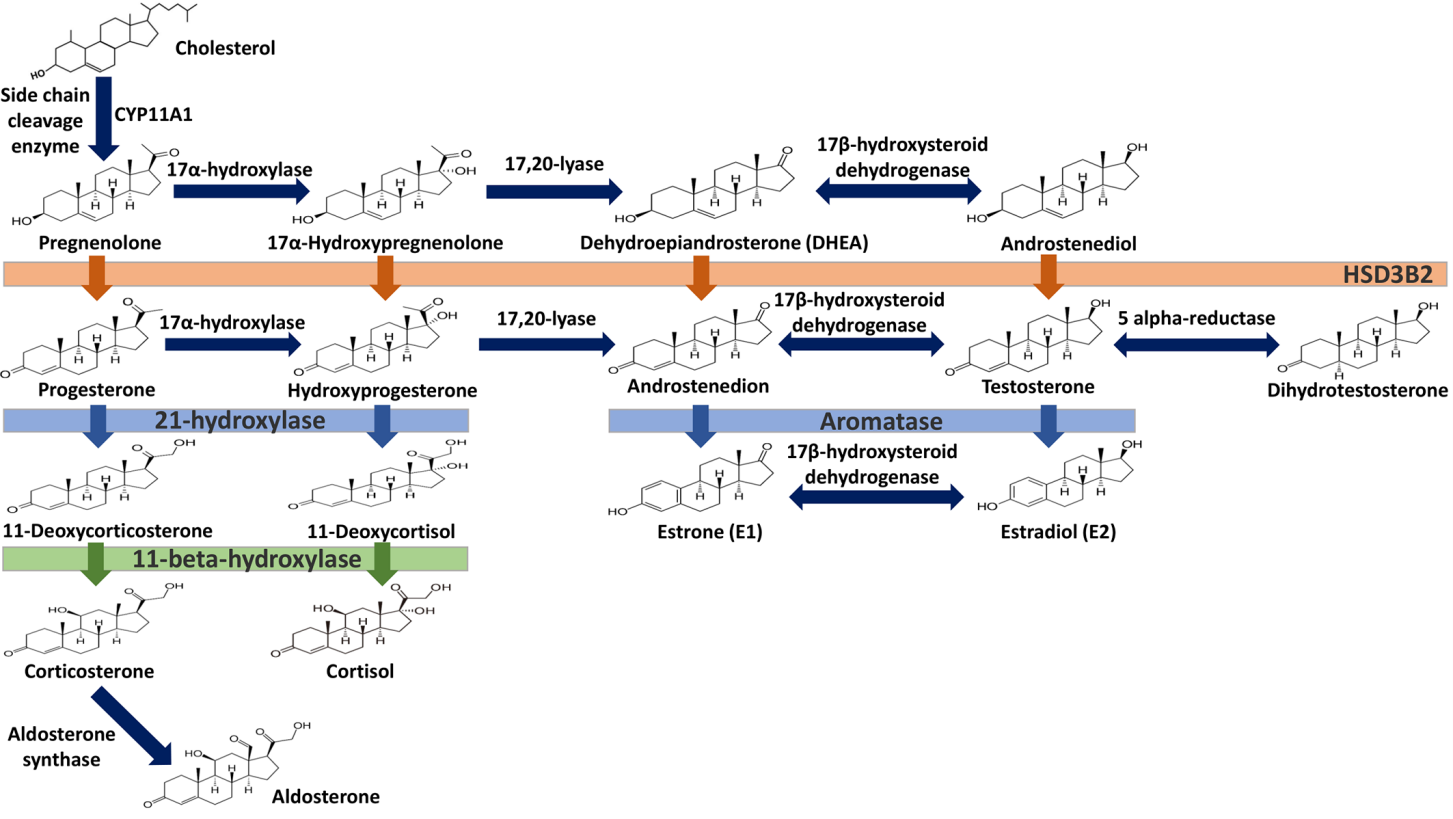
Steroid hormone synthesis and metabolism. The diagram designates key enzymatic steps in steroidogenesis.

**Figure 2:**
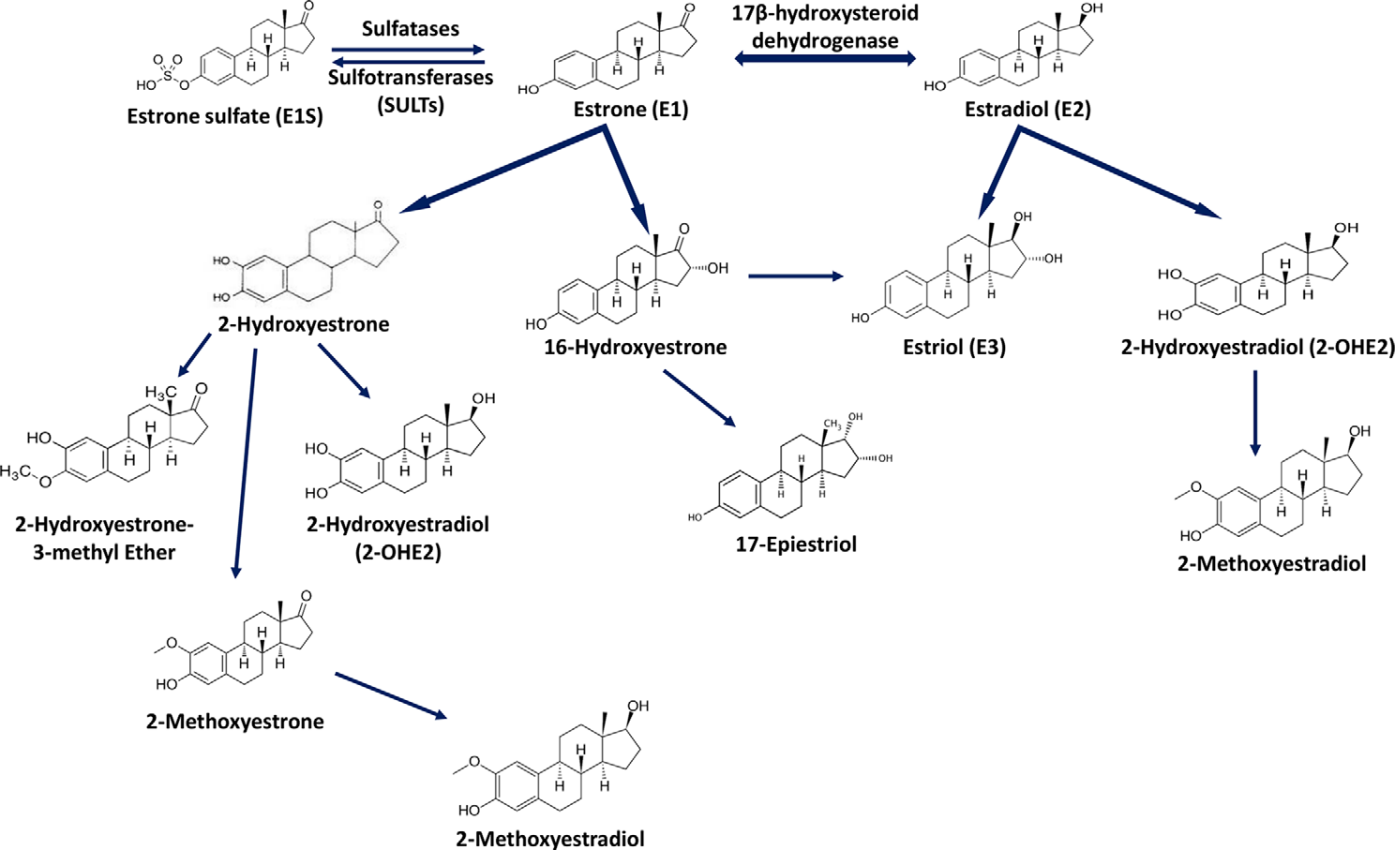
Estrogen metabolism. This schematic identifies key intermediates in the metabolism of estrone and estradiol.

Estrogens are gonadocorticoids and the primary female sex hormones. Their actions promote the development of female reproductive tissue and secondary sexual characteristics, and they influence all phases of reproduction including conception, fetal development, parturition, and nursing. Hence, estrogens exert their effects not only on reproductive tissue but on a wide range of physiological systems, including integumentary, central nervous, cardiovascular, skeletal, immune, metabolic, and excretory systems (1, 2). In humans, three forms of estrogen are synthesized. They are defined by their common 18 carbon (C-18) estrane ring structure and are numbered E1- E3 to reflect the number of hydroxyl groups linked to the estrane ring (**Figure 1**). Accordingly, they are named estrone (E1), estradiol (E2), and estriol (E3). Each of these endogenous estrogens is lipophilic and is presumed to exit and enter cells through their ability to freely diffuse across the plasma membrane. All endogenous estrogens are synthesized in the smooth endoplasmic reticulum in a shared pathway of steroidogenesis from cholesterol (C-27) (**Figure 2**). In this pathway, cholesterol is metabolized through a variety of enzymatic steps into (C-21) progestogens and (C-19) androgens that serve as the immediate steroid intermediate for estrogens. E1 and E2 are primarily secreted by ovarian granulosa cells in response to stimulation by neuroendocrine glycoprotein hormones, including luteinizing releasing hormone (LHRH), luteinizing hormone (LH), and follicle stimulating hormone (FSH), which are released from the hypothalamus and pituitary (3). During reproductive years, E1 and E2 are the two most common circulating estrogens found in plasma, with scant amounts of E3 measured. Estrogens can also be synthesized in a variety of non-ovarian tissues, including, adrenal gland, fat, brain, bone, skin, vascular smooth muscle and intestine (2). However, in these tissues, estrogens must be directly synthesized from androgens, as these tissues lack the necessary enzymatic machinery to synthesize C-19 androgens. E3 is synthesized at low levels in the liver and intestine by 16α-hydroxylation of E1 or E2 by cytochrome P450 enzymes, such as CYP3A4 (4). During pregnancy, E3 becomes the primary estrogen as it is synthesized at high levels by the placenta, far exceeding that of E1 or E2 in plasma. While its role in fetal development is not clear, low levels of E3 in maternal serum or urine is prognostic of poor perinatal health and congenital anomalies (5, 6).

The process by which estrogens are transported throughout the body and exert their biologic effects in target tissues is not completely understood. The vast majority of synthesized estrogen circulates in the plasma bound to either serum albumin or sex hormone binding globulin (SHBG) (7, 8). Only a small fraction (~ 1 to 2%) is unbound or “free” and available to bind to its receptors (9). E1 and E3 each bind SHBG with much lower affinity than E2 and likewise each of these estrogens also shows a much lower affinity and potency for its receptors than E2 (10). SHBG also binds dihydrotestosterone (DHT) and testosterone (T) but with relative binding affinities that are 20- and 5-fold higher than for E2 (11, 12). In premenopausal women, SHBG levels are twice as high as in men and this has been suggested to limit their androgen and estrogen exposure (9, 13). SHBG concentrations decrease following menopause but increase during the sixth decade of life (14), and low serum levels of SHBG have been associated with hyperandrogenism and endometrial cancer (13). Ultimately, estrogens are eliminated from the body following their metabolic conversion to inactive metabolites, which poorly bind SHBG, and are excreted in urine and feces. Metabolic conversion occurs primarily in the liver but also in other tissues, and involves their biotransformation *via* enzyme-mediated conjugation to glucuronide, glutathione, methyl, and/or sulfate moieties, modifications which enhance their solubility in plasma and enhance its absorbability by tissues (15) (**Figure 2**). Among these estrogen conjugates, estrone sulfate (E1-S) is the most predominant in plasma, and its reclamation by steroid sulfatase is yet another route by which estrogen biosynthesis may occur in extragonadal tissue (16).

**Figure 3:**
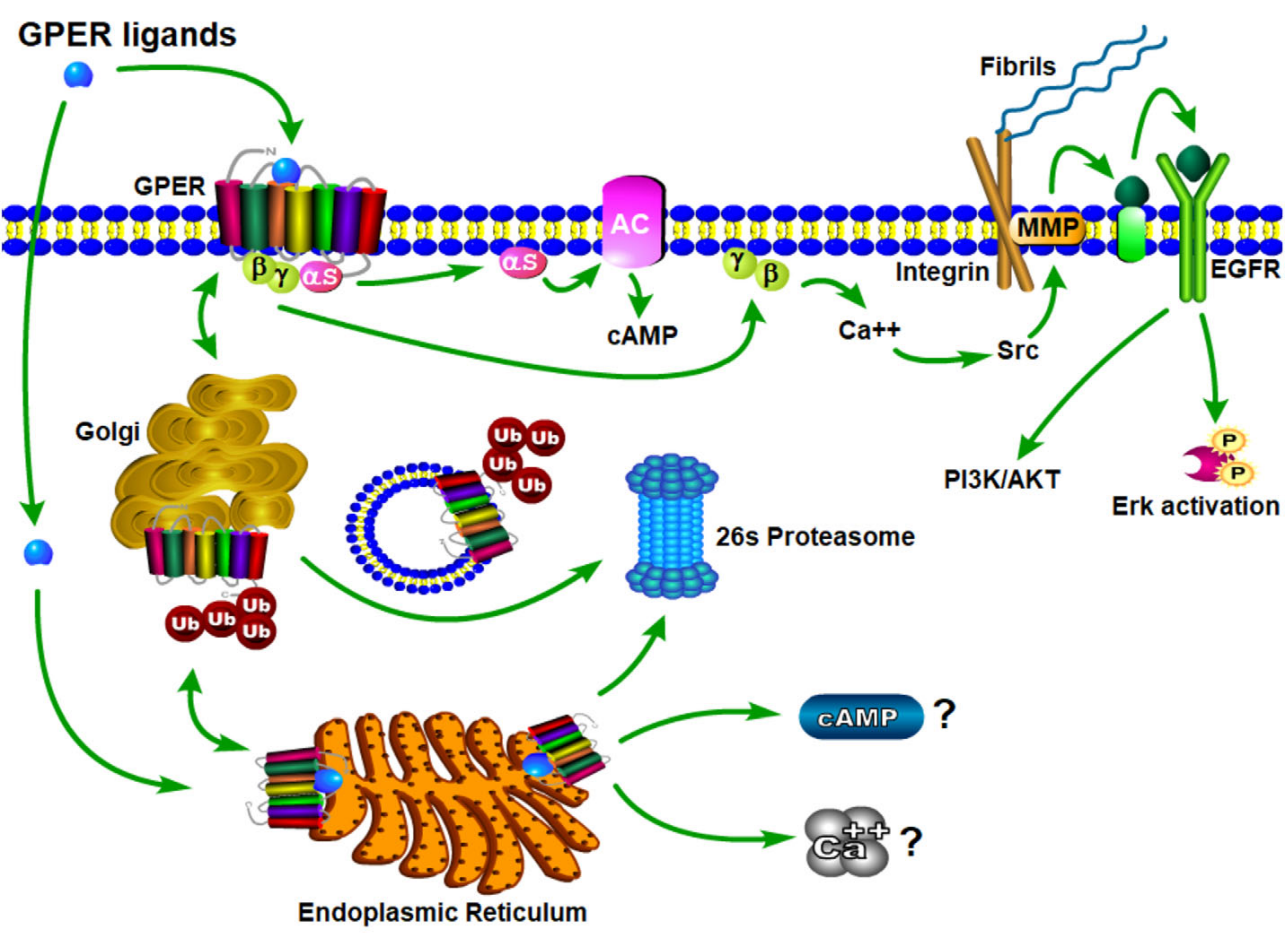
Schematic model of GPER trafficking and signaling. Nascent GPER is biosynthesized in the endoplasmic reticulum (ER) where it undergoes carbohydrate addition, editing and dimerization prior to forward trafficking through the Golgi apparatus during its transport to the plasma membrane. Misfolded GPER is polyubiquitinated and degraded at the 26S-proteasome. At the plasma membrane GPER exists as a high affinity GDP-coupled Gαβγ heterotrimer. Upon engagement of estrogenic ligands, GPER assumes an activated confirmation resulting in the dissociation of Gαs and Gβγ subunit proteins, which in turn, stimulate adenylyl cyclase and integrin-dependent release of membrane-tethered EGF-ligands, respectively. Independent studies evaluating retrograde trafficking of GPER suggest that it undergoes constitutive endocytosis and degradation *via* a ubiquitin-transGolgi-proteasome pathway. It is not yet clear whether sustained GPER signaling is observed from intracellular receptor (question marks).

The physiological effects of estrogen are manifested through the integrated action of cellular receptors that belong to the nuclear steroid hormone receptor (SHR) and G-protein coupled receptor (GPCR) superfamilies. This paradigm of coordinated signaling by estrogen through SHRs and GPCRs is evolutionarily conserved (17) and is also employed by progestogens (18, 19) and androgens (20). ER and GPER transmit intracellular signals *via* fundamentally distinct mechanisms that occur with distinct kinetics and involve unique signaling effectors (21) (**Figure 3**). In general, ERs are localized intracellularly and function as estrogen-inducible transcription factors, while GPER exhibits all the hallmarks of a plasma membrane receptor that manifests its actions through heterotrimeric G-proteins, which in turn transactivate plasma membrane receptors and enzymes (22). Evidence also exists that ERs may function similarly to GPER, and this has been reviewed elsewhere (23). Despite their differences in cellular location and mechanism of action, SHRs and GPCRs each undergo allosteric modulation in response to binding their cognate ligands, with signaling activity of SHRs and GPCRs enhanced by the physical interaction of their cognate ligands at specific receptor contact sites. The estrogen binding characteristics of GPER and ER are distinct, and they demonstrate a different dissociation constant, K_d_, in radiotracer assays using ^3^H-estradiol (**Table 1**). As discussed in detail (30), it is important to recognize that the relative binding affinities (RBAs) of ERα, ERβ and GPER cannot be readily compared due to the fact that ERs and GPER are expressed at different levels and they exist in different physicochemical environments; ER isolated in detergent-free cytosolic homogenates versus GPER enriched in lipid-rich plasma membrane preparations. Thus, the lower K_d_ that is measured for E2 in ER binding assays relative to GPER binding assays does not suggest that E2 has a higher affinity for ER relative to GPER. Because SHRs are readily isolated from the soluble fraction of cellular homogenates, crystallization and identification of physical ligand contact sites encoded with the structure of SHRs has been achieved (31–33). Crystal structures at resolutions of 2.6 angstroms for ER liganded to E2 or the ER antagonist, raloxifene (RAL), have been determined (34). These results show that E2 and RAL share contact sites with different binding modes and that each induces distinct conformations within the ER transactivation domain. The findings from these studies illustrate that the principal ligand contact sites of ER are defined within a hydrophobic cavity consisting of twelve helices (H1-12). Recognition of E2 within the ligand binding domain is achieved through a combination of hydrogen bond formation by the phenolic hydroxyls with polar residues contained within H3. H6 and H11, as well as alignment of the nonpolar character of estrane ring with hydrophobic residues that comprise these helices. As GPCRs are integral membrane proteins, purification is more challenging, and crystallization of GPER has yet to be achieved. In some regards, the hydrophobic environment provided within the closely aligned seven transmembrane helices of GPER is somewhat similar to the structure of the ER ligand binding domain. Several studies relying upon *in silico* molecular docking simulations have calculated principal binding interactions within the exoplasmic and/or transmembrane of GPER (35, 36). However, the role of these predicted ligand contact sites still needs to be evaluated by genetic studies which examine the influence of amino acid substitutions on GPER binding and signaling activity.

**Table 1:** Relative binding affinities of estrogenic ligands to estrogen receptors.

Ligand	Structure	Relative Binding Affinity (RBA)		
		ERα	ERβ	GPER
**Steroids**				
17β-estradiol (E2)	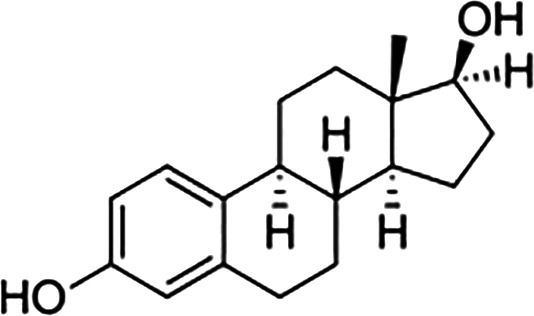	100	100	100
Estrone (E1)	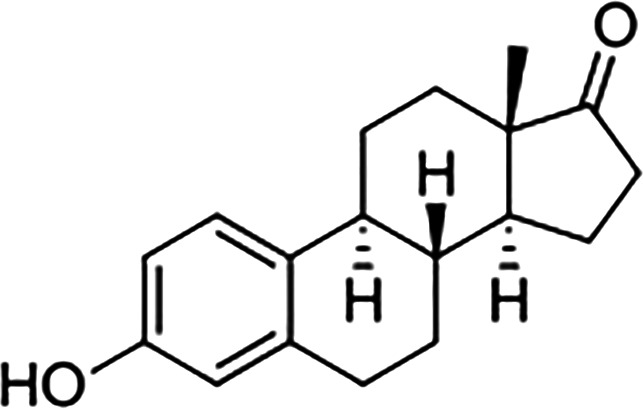	60	37	<0.04
Estriol (E3)	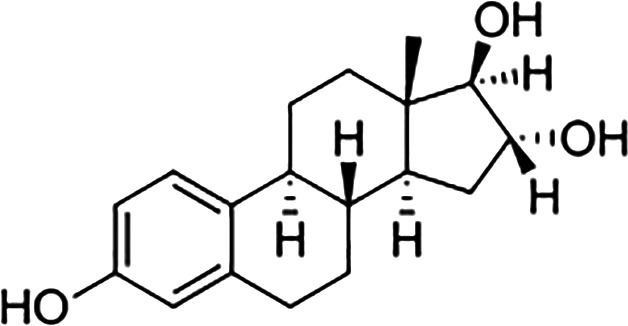	14	21	<0.4
17α- estradiol	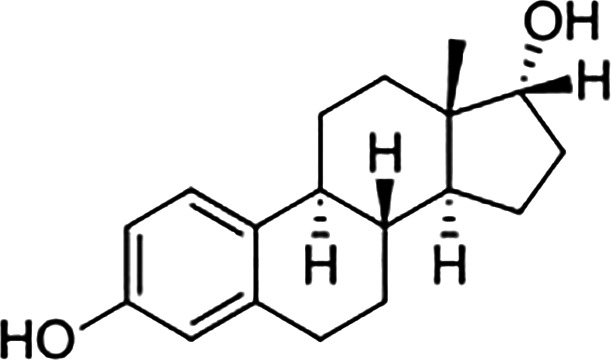	7	2	<0.04
Aldosterone	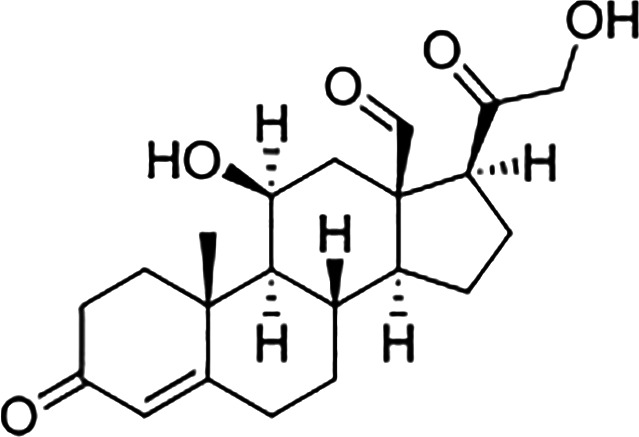	<0.0001	<0.0001 a	<0.00001
Diethylstilbestrol	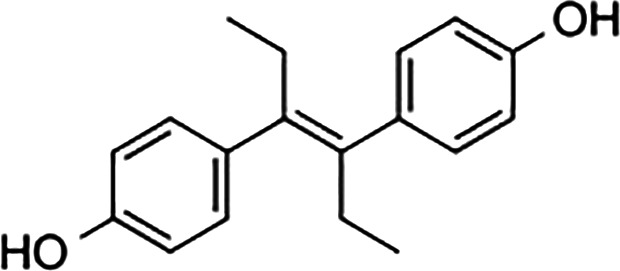	236	221	<0.4
4-OH-tamoxifen	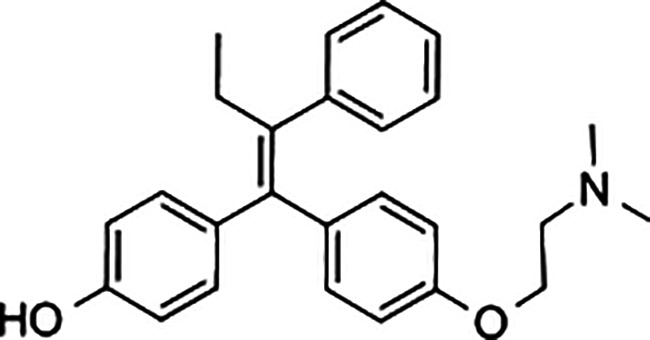	257	232	<4
**Man-made estrogens**				
Bisphenol A	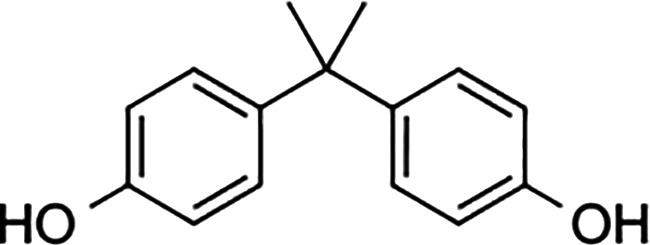	0.01	0.01	1.1^
Bisphenol S	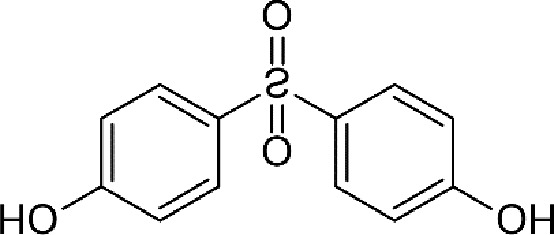	0.001	–	0.6^
Bisphenol F	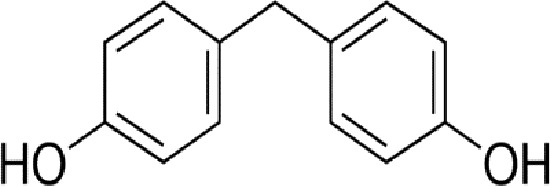	0.001	–	ND^
OH-PCB-4	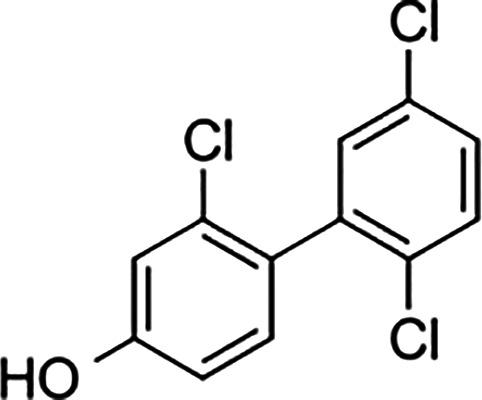	0.01	<0.01	0.1
p,p′-DDT	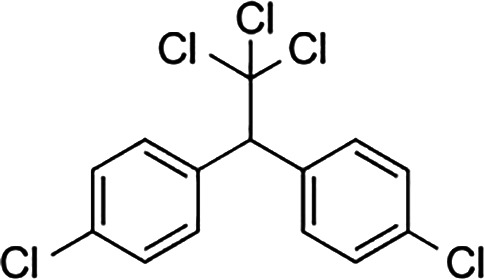	<0.01	<0.01	0.14
**Dietary estrogens**				
Genistein	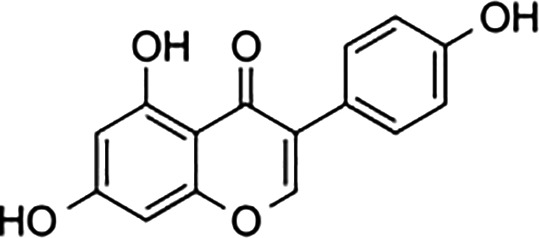	0.7	13	3
Zearalenone	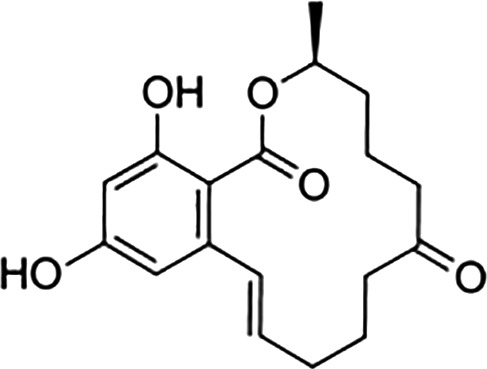	10	18	0.5
**Ligand**	**Structure**	**EC_50_ (nM)^#^**		
Daidzein	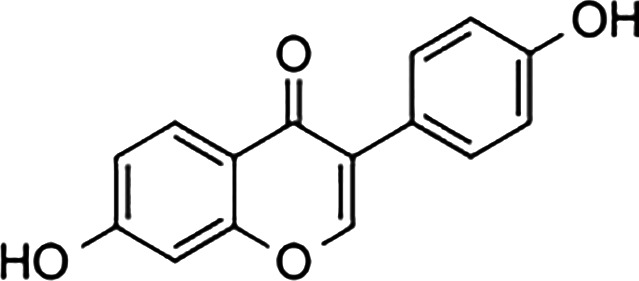	250	100	<1
Equol	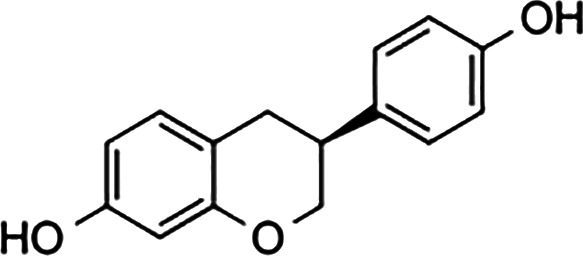	200	74	100

RBAs for ERα and ERβ are based on reports from multiple sources (24–28). RBA determined from solubilized receptor competition experiments. ^Data are based on fluorescence competitive binding assay. RBA for GPER are based on values taken from (29). **^#^**EC_50_ is calculated based on functional assays.

## GPER in Metabolic Disease and Cancer

Studies using knockout mice indicate that ER and GPER play different roles in estrogen physiology, with ER or GPER null mice primarily exhibiting reproductive (37, 38) and metabolic (39) deficits, respectively. This simple dichotomous description clearly oversimplifies the influence of each receptor type on estrogen physiology. However, collectively, the phenotypes of ER-null (40) and GPER-null (41) mice reflect the loss of reproductive function and metabolic homeostasis that is attributed to decreased ovarian estrogen biosynthesis accompanying menopause. While it is well appreciated that the metabolic effects of estrogen are manifested through ERs (42) and GPER (43), preclinical results published earlier this year in a study led by Sharma and Prossnitz, showed that chronic administration of the synthetic GPER selective agonist, G-1/Tespria, could restore fat, glucose and lipid homeostasis (44). This result indicates that targeting GPER may be an effective means for treating diabetes and obesity, and extends prior work that showed G-1 can ameliorate atherosclerosis in mice (45). The observation that chronic GPER signaling may alter metabolic activity has potential significance regarding a role for GPER in cancer as prolonged, uninterrupted estrogen exposure (46) and metabolic syndrome (47) are independent risk factors for cancer. Thus, GPER may serve as a centrally positioned factor that drives estrogen-induced carcinogenesis through chronic signaling that promotes metabolic disorder. In support of this concept, studies have linked GPER expression to clinical indices that predict advanced disease in breast cancer including increased tumor size, the presence of distant metastases, and tamoxifen-resistance (48–51). Similar results have been obtained in ovarian (52), endometrial (53), and testicular cancers (54) with GPER directly linked to poor survival. However, other reports suggest an inverse relationship between GPER and cancer progression (similar to that demonstrated by ER) (55, 56). The most likely explanation for the differences observed in the analysis of human cancer and GPER resides in the lack of a standardized procedure for its immunohistochemical detection and quantification in tumor biopsy specimens. For instance, some studies have set an absolute threshold for GPER expression among tumors, while others have focused on the relative difference between GPER in tumors and adjacent normal tissue in individual patients (55, 57, 58). Neither have laboratory studies resolved whether GPER is pro-oncogenic. Several observations strongly support that it is. First, GPER is required for the survival of xenograft-derived cancer stem cells and metastatic disease (59). Second, in breast cancer cells, GPER integrates assembly of the fibronectin matrix (60) with the release of EGF (61); thus satisfying two basic requirements or cellular survival: attachment to the extracellular matrix and responsiveness to growth factors. Third, in a preclinical model, breast cancer is less aggressive when GPER is genetically inactivated (62). Finally, the GPER selective antagonist, G36, delays the growth of type II endometrial cancer in mice (63). Nevertheless, other studies have suggested that GPER is tumor suppressive (64, 65). Specifically, stimulation with GPER-selective agonist, G-1 leads to pro-apoptotic signaling, as well as decreased proliferation and migration by cancer cells. Limitations of the latter studies are that G-1 was used at a 100-fold higher concentration than its reported K_i_ or EC_50_ (65) and receptor knockdown strategies were not used to test for off-target effects. In addition, these studies did not determine whether the G-1 responses also occur when the endogenous estrogen, E2 is applied, or for effects of selective GPER antagonists (G15 or G36). The latter point is particularly relevant because studies reporting GPER as tumor suppressive measured inhibitory biological responses. Other studies have reported that the GPER promoter is methylated in a small percentage of cancer biopsies (66). Then again, genetic silencing is observed for many genes in cancer specimens, and this could be explained by genomic instability. Indeed, promoter methylation of ESR-1 (ERα) is common in breast cancer (67, 68). Notably, epigenetic silencing of GPER as an anti-cancer mechanism is at odds with data in public repositories, showing that GPER is widely expressed, and rarely mutated, in solid or hematopoietic cancers and in cancer cell lines. Thus, the conclusion that GPER is “tumor suppressive” is inconsistent with the widely accepted concept that a tumor suppressor gene requires genetic inactivation or epigenetic silencing. Furthermore, the idea that GPER is anti-oncogenic does not fit well with findings which suggest an active role for GPER in cancer progression in the tumor microenvironment (21). Specifically, the hypoxic environment created by proliferating cancer cells favors increased expression of GPER and local estrogen production. Breast cancer cells and cancer-associated fibroblasts (CAFs) upregulate GPER expression *via* hypoxia-inducing factor-1α (HIF-1α)-regulated transcriptional control (69). Increased AP-1 mediated aromatase transcription and activity is measured in breast cancer cells following estradiol or tamoxifen-mediated stimulation of GPER (70). Nor does an anti-oncogenic role for GPER reconcile with bioinformatic analyses that show that its expression correlates directly with pro-metastatic signaling pathways in estrogen receptor negative breast cancer (71). Nevertheless, the discrepancy between the pro-oncogenic and tumor suppressive activities of GPER has been discussed (72) and underscores the need to define the mechanisms that drive GPER activity and their relationship to oncogenesis.

## Implications of GPER for Anti-Estrogen Therapy

ERs and GPER act independently but coordinately to maintain homeostasis of estrogen-responsive tissue. Thus, it is likely that neoplasms that arise from these tissues may either continue to direct estrogen action through both receptor types or lose control of one or both receptor mechanisms during their evolution. In fact, this is the pattern that is observed in breast cancer with treatment-naive tumors containing both receptors, one or the other receptor, or neither receptor (73). From a clinical perspective, GPER disrupts the ER-centric, binary rubric which categorizes breast cancer as either estrogen responsive or nonresponsive, with nearly, 20% of all breast cancers expressing GPER in the absence of ER. Interestingly, a preponderance of these ER-GPER+ tumors are triple negative breast cancers that lack ER, progesterone receptor (PR) and her2/neu (74).

Therapeutic interventions that reduce bioavailable estrogen should be an effective means to prevent the biological action of ERα, ERβ, and GPER. At present, three common methods are employed for reducing bioavailable estrogen: i) ovarian ablation by ovariectomy or radiation, ii) ovarian suppression by bolus administration of a gonadotrophin releasing hormone (GnRH) superagonist, such as goserelin or leuprolide, or iii) chemical inhibition by administration of aromatase inhibitors (AIs), such as exemestane, letrozole or anastrazole. Each of these three treatment interventions are used for the treatment of breast cancer. However, no single method for reducing estrogen is failproof and each of these approaches induces premature menopause, which is associated with long-term mortality risks, including increased risk of cardiovascular disease (75) and loss of bone density (76), as well as menopausal symptoms that can impact on quality of life (77). Elimination of ovarian function, either permanently by ablation or temporarily by interrupting the neuroendocrine circuit of estrogen biosynthesis, does not interfere with nonovarian biosynthesis. AIs are effective in this manner in that their effects prevent estrogen biosynthesis independent of tissue origin. While AIs effectively delay breast cancer progression in approximately 50% of breast cancer patients, their beneficial value in the remaining patients is offset by their high rate of acquired and *de novo* resistance (78). In evaluating the efficacy of blockade of estrogen biosynthesis in the context of either GPER (or ER), it is important to point out that nuclear steroid hormone receptors (SHRs) and G-protein coupled receptors (GPCRs) are allosterically regulated receptors that are capable of ligand-independent action (79, 80). Thus, inhibition of estrogen biosynthesis may not be effective for patients whose tumors contain mutant receptors that lose ligand binding activity but retain constitutive signaling. Although ligand binding mutants have not yet been defined for GPER, they have been identified for other GPCRs (81) and for ER (82).

An important concern regarding therapies that block estrogen biosynthesis is that theoretically they should effectively increase the ability of exogenous estrogens to interact with their cellular receptors. Albeit, it is not known whether or not AIs alter the interaction of exogenous estrogens with either GPER or ER, as this has not yet been tested experimentally. This idea is particularly interesting in light of the fact that although xenoestrogens show low binding affinities relative to 17β-estradiol for ER. The same is not true for GPER, as xenoestrogens show much higher relative binding affinities for GPER (**Table 1**). In order to illustrate their potential effect on anti-estrogen therapy in the context of GPER, a few of the more abundant exogenous estrogens that are relevant for this discussion are mentioned here. For example, in independent assays, the dietary soy isoflavone, daidzein (DZN) exhibits a high relative potency for GPER relative to ER, with an EC_50_ in the subnanomolar range compared with an EC50 that is more than 100- to 200-fold higher for nuclear ERs. Dietary exposure to soy is not trivial, in fact, measurements of postprandial serum concentrations of DZN can exceed preovulatory levels of E2 by 10-fold (83). Adding further complexity to the influence of phytoestrogens on breast cancer is the popular belief that a soy-rich diet is breast cancer protective (84). Epidemiological studies have placed emphasis on whether metabolism of DZN to S-equol, which is exclusively mediated by the gut microbiome is a critical factor in influencing estrogen physiology and ER-targeted therapy (85). This concept is interesting in light of the finding that Eastern women, whom show a two-fold reduced risk for developing breast cancer relative to Western women are twice as likely to harbor gut bacteria that metabolize DZN to S-equol (86). However, the oncogenic activity of DZN and S-equol is unclear as DZN exerts pro- and anti-oncogenic activity in mice, while other studies suggest that S-equol is anti-oncogenic (84). The influence of dietary estrogens on estrogen-targeted therapies is controversial (87). A recent guidance statement from the American Association of Clinical Endocrinologists (AACE) suggests that a soy-rich diet may be used as an alternative approach for estrogen replacement therapy (88) indicating that endogenous estrogens and phytoestrogens are biologically equivalent. Yet, an oft quoted study of 524 postmenopausal Chinese women with breast cancer showed improved survival and less recurrence in patients with the highest quartile of soy intake relative to counterparts in the lowest quartile of soy consumption (89). Significantly, this study showed a significant risk increase for patients receiving tamoxifen compared to those that received anastrozole. These data have been interpreted to indicate that soy may act competitively to block binding of tamoxifen to ER. Alternatively, these findings may suggest that the poorer survival observed in the tamoxifen arm of the study may be due to the fact that tamoxifen and soy isoflavones function as GPER agonists. Moreover, the Kang study did not control for obesity nor bacterial metabolism of DZN. Nonetheless, in humans avoidance of dietary soy or ingestion of DZN supplements by breast cancer patients receiving estrogen targeted therapy is encouraged (90) despite the fact that the RBA of DZN is 0.003% for ERα and 0.05% for Erβ (91). The question of whether soy isoflavones show enhanced carcinogenicity in the absence of endogenous estrogen has not yet been carefully addressed. Human and mouse studies which control for phytoestrogen intake, gut metabolome, and obesity in the presence or absence of AIs are necessary to evaluate the carcinogenicity of soy isoflavones in the face of AI therapy.

GPER also provides similar concerns regarding the carcinogenicity of the plasticizer, bisphenol A (BPA), the highest volume chemical produced world-wide (92). Human exposure to BPA is significant as >90% of the US population contains measurable amounts of BPA, with highest levels in children (93). BPA exhibits an RBA for GPER that is 100-fold greater than that measured for nuclear ERs (**Table 1**). In vitro studies indicate that BPA potency for GPER is high, with biological effects measured in the low nanomolar range in breast cancer cells and breast cancer-associated fibroblasts (60, 94) and in human seminoma and testicular cancer cells (95). Exposure to BPA is associated with many human diseases, including obesity, diabetes and cancer, and is able to induce toxicological effects in tissues and cultured cells (96). The Environmental Protection Agency and the Food and Drug Administration agree upon a safe reference dose (RfD) for BPA in humans at 50 μg/kg/day that was scaled from toxicology studies in rodents (97). Carcinogenicity testing at doses below and above the RfD in mice has yielded mixed results. While BPA is not considered a robust carcinogen, early life exposures in rodents at the RfD is associated with prostate and breast cancer (98). These authors duly underscore that the most vexing variable in the analysis of BPA carcinogenicity is the acknowledged error of scaling RfD between man and rodent due to the fact that BPA exhibits nonmonotonic dose responses in many biochemical and biological assays (99). Even more significant with regards to GPER, urinary concentrations of BPA in participants in the National Health and Nutrition Examination Survey (NHANES) demonstrated a positive association with metabolic syndrome (100). Moreover, exposure to BPA correlates with an increase in serum SHBG, even though BPA shows poor binding affinity for SHBG (12). Thus, theoretically, for a patient receiving AIs, BPA is a particularly potent GPER agonist. However, this has yet-to-be addressed in studies in which dietary estrogen intake, obesity, and gut metabolome are carefully controlled. Nonetheless, BPA is a particularly troubling environmental estrogen due to the fact that it is a malleable chemical structure that has been manipulated by chemists to produced more than 40 analogues. Many of these BPA similar are detected in humans at even higher concentrations than BPA (93, 101), and at least seven BPA analogues exhibit similar RBAs and relative potencies for GPER in breast cancer cells (35).

ER antagonism, using a selective estrogen receptor modulator (SERM), such as tamoxifen or a selective estrogen receptor degrader (SERD), such as fulvestrant, is yet another form of anti-estrogen therapy that is widely effective in the treatment of breast cancer, providing greater than 10 year survival in postmenopausal women with early stage, ER-positive cancer (102). Still, not all of these patients respond to ER antagonists, as *de novo* resistance occurs, and this may be due to many reasons, including: i) the presence of constitutively active ER mutants, ii) hyperactive growth factor signaling, or iii) the presence of an alternative estrogen receptor, i.e. GPER (103). GPER adds further complexity to anti-estrogen therapy in that ER antagonists, including tamoxifen, faslodex and raloxifene function as GPER agonists (21, 29). Furthermore, ER antagonism or AIs are not effective for postmenopausal women with late stage disease or for premenopausal women (104). Consistent with this idea, results from the SOFT (Suppression of Ovarian Function Trial) suggest that even further supplementation of estrogen-targeted therapy (Tamoxifen or AI) by adding ovarian suppression for premenopausal ER-positive breast cancer, while effective in reducing serum estrogen and disease relapse had no effect on overall survival (78). In this study, patients were not further stratified by whether their tumors expressed GPER. However, an argument could be made that patients whose tumors lacked GPER [approximately one-third of ER+ tumors (73)] may be more likely to respond to ER antagonism plus ovarian suppression. Further confusion regarding the role of estrogen and its receptors in female reproductive cancer comes from the disconnect between menopausal status and proliferative index, as measured by Ki-67 in tumor biopsy tissue. Breast tumors from patients with intact ovaries, show high mitotic indices, while postmenopausal women with ER-positive breast cancer are assigned either anti-estrogen therapy regardless of Ki-67 index (105). Chemotherapeutic agents, which are toxic but target rapidly proliferating cells are layered on top of anti-estrogen therapy for patients with aggressive estrogen-dependent cancers (106), without consideration of their GPER status, which has been tied to chemotherapeutic resistance *via* its capacity to trigger EGFR transactivation (107). Recent results from the PALOMA-III trials, further showed that addition of palbociclib, which targets cyclin-dependent kinases, CDK4 and CDK6, to ER-targeted therapy (fulvestrant) provides increased overall survival for patients with advanced ER-positive breast cancer (108). Early results achieved with palbociclib in metastatic breast cancer are encouraging. Yet they do not resolve whether palbociclib selectively targets proliferation in fulvestrant- resistant, ER-positive breast cancer cells, or whether its actions directly influence GPER-dependent cellular responses associated with tumor cell metastasis and disease progression. Collectively, these examples indicate that definition of GPER status for patients with breast cancer may help to select patient populations which are best able to respond to existing anti-estrogen therapies, either ovarian suppression, ER antagonism or aromatase inhibitor.

## Existing and Future Pharmacological Compounds That Target GPER

For all of the above reasons, therapeutic approaches that block GPER action hold great promise for the treatment of cancer. After all, nearly one-third of all FDA-approved drugs target GPCRs (109). While GPCR targeted drugs have been predominately used for the treatment of cardiovascular disease and diabetes, the concept of developing GPCR targeted cancer therapeutics has gained traction over the past decade (110). This is largely due to preclinical studies which link GPCRs to cancer growth and metastasis, often in a scenario where the GPCR involved is chronically exposed to local or circulating agonist. Examples of this include, the bioactive lipid, lysophosphatidic acid, and its receptor, LPAR-1 in breast cancer (111), chemokines, CXCL8/IL8 and CXCR1 and CXCR2 in melanoma, pancreatic cancer and gastric tumors (112) and CXCL12 and CXCR4 in multiple cancers (113). Consistent with the notion that chronic estrogen exposure may drive GPER oncogenesis, breast tumors with increased GPER plasma membrane density show poor prognosis (51). This may be consistent with the concept that GPCRs often demonstrate a hyperbolic relationship between ligand occupancy and receptor response (114). This is widely described as “fractional occupancy” and suggests that a small change in GPER plasma receptor density could result in a more than linear increase in GPER activity. It is also important to consider that GPER shows specific binding activity to estrogenic ligands, natural or synthetic, which are hydrophobic and/or lipophilic and easily diffuse through or insert themselves into a lipid bilayer. In fact, it has previously published that crude membrane fractions exhibit specific GPER binding activity (Thomas et al, 2005). Whether intracellular interaction between GPER and its ligands allows for sustained intracellular signaling or plays a role in the proper folding and transport of GPER to the plasma membrane has not yet been determined. In this regard, it is important to recognize that an intracellular staining pattern is observed in most, but not all, cell types (115). However, a plasma membrane staining pattern by immunohistochemical (IHC) analysis of microtome-sectioned, archival paraffin-embedded tissue is not easily detected unless the majority of the receptor is at the plasma membrane, and little is detected intracellularly. With this in mind, slight differences in GPER ligand sensitivity would be difficult to detect by IHC, however, measurement of GPER plasma membrane density by flow cytometric analysis of intact breast cancer cells (116) may provide a better handle as whether to apply anti-estrogen therapy in the context of GPER-targeted therapies described below.

### Small Molecule GPER Antagonists

Several GPER antagonists have been developed (**Table 2**). While many of these first-generation drugs hold promise, we review below two GPER antagonists with half-maximal inhibitory concentration (IC_50_) within the nanomolar range. The first GPER antagonist in this class, named G15, was developed by Prossnitz and colleagues using a combination of virtual and biomolecular screening steps (117). First these authors used a software-assisted virtual screen of the NIH Molecular Libraries Small Molecule Repository (MLSMR) of 144, 457 molecules. From this primary screen 57 compounds were isolated that were similar in structure to the GPER selective agonist, G-1, a substituted dihydroquinolone (24). These compounds were tested subsequently for their capacity to inhibit E2-mediated calcium mobilization in human SKBR3 breast cancer cells that express endogenous GPER but lack ERα and ERβ. G15 emerged from this screen based on its: i) structure and presumed ability to interact competitively with E2, ii) ability to block E2-dependent calcium signaling, and iii) measured binding affinity (K_d_ = 20 nM) for GPER, which was assessed using I^125^-labelled G-1 as radiotracer. G-15 displays relatively low binding affinity for ERα and ERβ as measured in a competition assay employing an Alexa 633-estradiol conjugate as fluorotracer (K_i_ > 10 nM). *In vivo* testing has shown that G15 blocks a proliferative response in uterine epithelial cells (117). A G15 derivative, named G36 was subsequently synthesized by Dennis and Prossnitz, with even lower affinity interactions with ERα (118). G36 inhibits E2 and G-1-dependent calcium mobilization as well as erk-1/2 activation in SKBR3 cells (IC_50_ = 200 nM) and blocks the growth of transplanted estrogen-dependent type II endometrial cancer cells (63). Recently, Chris Arnatt and David Wang have collaborated to report a new GPER antagonist that protects ovariectomized ERα null mice from estrogen-induced cholesterol gallstones (119). Using a receptor-ligand interaction computational screen, a novel series of GPER-selective antagonists were generated, including one new compound, 2-cyclohexyl-4-isopropyl-N-(4-methoxybenzyl) aniline (CIMBA). that shows strong antagonism with selectivity for GPER. Specifically, CIMBA inhibits G-1 dependent calcium mobilization in HL60 cells (IC_50_ = 75 nM), with a binding activity for ERα or ERβ <10 μM in fluorescence polarization assays. Some differences were noted by Arnatt and colleagues with regards to the efficacy of G15, G36 and CIMBA to inhibit calcium mobilization, although all three GPER antagonists each showed inhibitory capacity for G-1 induced cAMP accumulation by homogenous time resolved fluorescence (HTRF). Thus, while the computational algorithms that yielded the G-series based and methoxybenzyl aniline based GPER antagonists were inherently distinct, both show similar capacity to inhibit G-1 induced GPER signaling, with each showing efficacy for reducing estrogen-induced pathology in mice.

**Table 2:** IC50 for GPER antagonists.

Ligand	Affinity	Reference
	IC_50_ (nM)
**G15**	*^a^*190 *^b^*185	(117)
**G36**	*^a^*112 *^b^*165	(118)
**CIMBA**	*^c^*60-90	(119)
**MIBE**	*^c^*1,750	(120)
**PBX1**	*^c^*250	(121)
**PBX2**	*^c^*300	(121)
**C4PY**	*^c^*900	(122)
**CPT**	5,000	(123)

^a,b,c^ IC_50_ was measured by competition binding assay to GPER between antagonist and fluorescent estrogen, iodinated G1 analog and [^3^H] E_2_, respectively.

### Targeting G Proteins

An alternative approach to developing selective agents that block GPER action is to employ pharmaceutical compounds that directly target G proteins (124, 125). This strategy has the added benefit that although GPER is a driving force in the genesis of metabolic disorder and cancer, these are complex diseases in which multiple GPCRs are involved. Primary examples include chemokine receptors (CXCR1, CXCR2, CXCR4, CCR5, CCR7) that drive chronic inflammatory responses common to both obesity and cancer. The premise by which G protein blockade is effective as a therapeutic is the ability of these agents to preferentially inhibit signaling pathways shared by more than one GPCR. Towards this end, cell permeant pharmacological agents have been developed that interfere with conformational activation of the GPCR-Gαβγ complex following ligand binding. To date, pharmacological compounds that specifically inhibit Gα-GTPase have been limited to the Gαq proteins and include YM-254890 (126) and FR900359 (127). Gαq inhibitors show good preclinical success in thrombosis (128), asthma (129) and melanoma (130). In contrast, Gβγ inhibitors, which were initially based upon the carboxyl terminal domain structure of G-protein receptor kinase 2 but now also include M119 and gallein, show efficacy in preclinical models of opioid analgesia, chronic inflammatory disease, heart failure (124). Blockade of GPER-dependent EGFR transactivation in breast cancer cells is effective using a Gβγ-sequestrant peptide (131), and further study is needed to evaluate whether Gβγ-inhibitors are effective in mouse models of metabolic disorder and cancer.

“Biased” agonists that stabilize a GPCR conformation that preferentially activates one signaling pathway over another (132) represents a related approach towards selective inhibition of G protein dependent signaling. Oliceridine, a biased agonist for μ-opioid receptor was developed to favor Gαi-inhibition of adenylyl cyclase over Gβγ-dependent activation of β-arrestin (133) and has been evaluated in clinical trials for chronic pain. Although recent reports indicate that low agonist efficacy, rather than receptor bias, may explain the low side effect profile of oliceridine (134). Similarly, biased agonists have been developed and characterized for angiotensin I receptors that preferentially recruit β-arrestin for their potential use in reducing hypertension (135). Biased agonists have yet to make their way into the clinic. However, it is unclear at the moment whether the biased agonist conformation is unique to certain GPCRs or whether it has broad application. Still, our environment is replete with compounds that function as estrogen mimetics, and it may be possible by high throughput analysis of synthetic and nutraceutical compounds to identify biased GPER agonists that may have therapeutic value.

### Targeting Downstream Signaling Effectors of GPER

Via GPER, estrogens trigger an epidermal growth factor (EGF)-autocrine loop (22) that holds significance for breast carcinoma, and potentially other malignancies that arise from epithelial tissue. In breast cancer this holds particular significance due to the reciprocal relationship that is often observed between ER and epidermal growth factor receptors (EGFRs) in primary tumors. This relationship has fostered the dichotomous categorization of breast cancers as either estrogen responsive or growth factor responsive. While GPER disrupts this simple binary scheme, GPER holds potential diagnostic value in selecting patients that may best benefit from either erbB1 or erbB2/her2/neu targeted therapy, particularly among premenopausal women. Assessment of GPER expression also may suggest the appropriate combinatorial assignment of AI or GPER antagonist with EGFR targeted antibody treatment. As discussed in section 3, GPER is expressed in a majority of TNBC, an aggressive subtype of breast cancer with no known molecular targets. erbB1/EGFR is also commonly overexpressed in TNBC, although results from numerous clinical trials reveal low response rates to anti-EGFR therapy for patients with TNBC (136). However, some patients do respond well, which may suggest a need to stratify patients for EGFR responsiveness and to develop combinatorial therapies. In both regards, GPER may have value. First, as a theranostic index. Second, GPER targeted therapeutics may fit well as part of a combinatorial anti-EGFR therapy for patients with erbB1 overexpressing TNBC.

Phosphoinositide 3 (PI3) kinase/AKT signaling lies downstream of erbB1/erbB2, and is activated following GPER stimulation (137). Activation of PI3K/AKT signaling occurs commonly in breast cancer and is associated with endocrine resistance and worse prognosis (138). Pan-PI3K inhibitors have fared poorly in clinical trials due to their toxicity, while the isoform-specific PI3K inhibitor, alpelisib, has been approved by the FDA as co-therapy with fulvestrant for patients with ER-positive, PI3Kalpha mutated advanced breast cancer (139). FDA approval of alpelisib with fulveestrant followed the results of the SOLAR-1 trial that showed that patients receiving alpelisib with fulvestrant showed a median increase of 6 months of progression free survival. Future studies that include a more comprehensive view of patients which are estrogen responsive by including analysis of GPER, may lead to similarly designed clinical trials that combine either AIs or GPER targeted therapy with alpelisib.

### Antibodies

Traditionally, small molecules have dominated as the preferred means to target GPCRs but recent pharmaceutical trends that favor immunotherapeutic approaches have led to the development of GPCR-targeted antibodies for clinical use (**Table 3**). The most significant progress has been made in the development of antibodies that block the binding of chemokines to their cognate GPCRs in cancer and inflammatory disease (144, 145). Notably, mogamulizumab/Poteligeo, an anti-CCR4 targeted therapy for refractory adult T cell leukemia and mycosis fungoides has received FDA approval (146). Likewise, the FDA has also approved erenumab/Aimovig, targeting Calcitonin Gene-Related Peptide Receptor (CGRPR) as a prophylactic treatment for migraine headaches (141). In addition, the angiogenesis/tumor metastasis-associated receptor, CXCR4, targeted by ulocuplumab (Bristol Myers Squibb), a fully humanized antibody that blocks binding of stromal-derived factor 1 (SDF-1) in adult myeloid leukemia has entered phase II trials (143). The CCR5-targeted antibody, leronlimab is currently under phase III investigation as an HIV therapy and has entered phase II testing to relieve chronic lung inflammation that accompanies COVID 19 infection (142). CCR2 targeted mAB, MLN1202/plozalizumab (Millenium/Takeda Oncology) has been evaluated in multiple clinical trials for cancer and other indications (147).

**Table 3:** Status of GPCR therapeutic antibodies.

GPCR	Drug name	Brand name	Status	Indication	References
CCR4	mogamulizumab	Poteligeo	Approved, 2018	mycosis fungoides Sezary syndrome	(140)
CGRPR	erenumab	Aimovig	Approved, 2018	migraine prophylaxis	(141)
CCR5	leronlimab		Phase III Phase II	HIV COVID-19	(142)
CXCR4	ulocuplumab		Phase II	multiple myeloma	(143)
CCR2	plozalizumab		Investigational	diabetic nephropathy	

Once considered difficult to target *via* antibody-based approaches, the combined use of lipid-enriched GPCR preparations and the development of recombinant phage display technology has allowed for the rapid growth and development of antibodies that target GPCRs. The fact that GPCR heterodimerization is a widely accepted paradigm that adds diversity and complexity to GPCR functionality is an additional reason why antibody-based therapeutic approaches have gained traction relative to small molecule antagonists.

Antibodies that target GPCRs could also be used to deliver anti-cancer agents by conjugating the antibodies to nanoparticles (148). Such nanoparticles can be formed from biodegradable polymers and can physically entrap the anti-cancer agent throughout the nanoparticle (149). Through diffusion and degradation of the polymer, the drug can be released in a controlled manner to the target cancer (149). Polymers used to prepare these particles include but are not limited to poly lactic-co-glycolic acid (149), polysulfenamides (150), and polyanhydrides (151). Agents that can be loaded into the particles include proteins such as cancer antigens (152), nucleic acid based molecules like plasmid DNA (153) and CpG (154) and small molecule drugs like paclitaxel and doxorubicin (149, 155).

## Conclusions

Anti-estrogen therapies are successfully employed for the treatment of breast cancer and anovulatory infertility. Still, at present, decisions regarding the appropriate assignment of anti-estrogen therapy in breast cancer are limited strictly upon the detection of ER in tumor biopsy specimens. This ER-centric perspective ignores the fact that 20% of breast cancers express GPER and in the absence of ER (73), and that GPER is expressed in a majority of TNBCs (74). Despite the relative success of ER antagonists, aromatase inhibitors and ovarian ablation/suppression strategies for postmenopausal women with early stage ER- positive cancer, resistance occurs. A further confounding variable for the assignment of anti-estrogen therapy is the fact that ER antagonists (both SERMS and SERDs) function as GPER agonists, which aligns with the finding that GPER is associated with tamoxifen resistance in breast cancer patients (103). The realization that daidzein (156) and environmental bisphenols (35) potently activate GPER further alters our perspective regarding the appropriate assignment of anti-estrogen therapy. In addition, recent clinical trials evaluating AI or TAM with ovarian suppression have shown a median increase in progression free survival suggesting that some patients may respond favorably to tandem anti-estrogen blockade. However, these studies did not include patients whose tumors are GPER- positive and ER-negative. Our current perspective for determining which patients may respond to anti-estrogen therapy is evolving, and is bolstered by findings that show that GPER associates with cancer progression variables (48, 52, 53), activates cellular receptors that facilitate cancer cell survival (54), promotes the survival of patient-derived breast cancer stem cells (59), and acts in the tumor microenvironment to drive cancer metastasis (62).

The development of GPER targeted therapies holds the promise of expanding our existing arsenal of estrogen-targeted therapies. GPER is a therapeutic target that holds particular promise for the treatment of several critical health concerns facing Western society, including obesity, diabetes, vascular pathology and advanced cancer. In the preclinical setting, chronic administration of G1/Tespria restores fat, lipid, and glucose homeostasis in obese and diabetic mice without uterotropic effects (44). Analysis of human cancer and in mice suggest that GPER is linked to advanced cancer, and chronic estrogen exposure and metabolic syndrome are independent risk factors for many cancers. Thus, GPER provides a likely mechanism by which metabolic disorder may be part of the landscape for estrogen-driven malignancies. The selective GPER antagonists, G15 delays the growth of endometrial cancer (63) and exciting new data indicates that a new GPER antagonist, CIMBA, can prevent estrogen-induced gallstones (119). Additional methodologies for targeting GPER may also include direct blockade of G-proteins, the development of biased agonists and therapeutic antibodies. Collectively, these approaches may complement existing anti-estrogen therapies and improve our approach towards treating patients suffering from estrogen-driven malignancies and disease.

## Author Contributions

All authors contributed to the article and approved the submitted version.

## Funding

EJF and AKS acknowledge the NIH NCI P30 CA086862 Cancer Center support grant as well as I-award funds from the Holden Comprehensive Cancer Center, including an Oberley award and monies from the Breast Cancer Research Interest Group. EJF acknowledges funds from the Department of Surgery at the University of Iowa. AKS is the Lyle and Sharon Bighley Chair in Pharmaceutical Sciences.

## Conflict of Interest

The authors declare that the research was conducted in the absence of any commercial or financial relationships that could be construed as a potential conflict of interest. 
